# A Preliminary Examination of the Associations Between Sleep Quality and Body Dysmorphia Symptoms Among Two Separate Samples of Adolescents

**DOI:** 10.1007/s10578-025-01813-7

**Published:** 2025-02-11

**Authors:** Sarah A. Bilsky, Leila C. Sachner, Racheal A. Embry, Kayce M. Hopper

**Affiliations:** Department of Psychology, University of Mississippi, 302B Peabody Hall, Oxford, MS 38655 USA

**Keywords:** Sleep quality, Adolescent, Body dysmorphia, Anxiety, Depression

## Abstract

Obsessive compulsive and related disorders have been linked to sleep disturbances. Given that adolescence is a core risk period for body dysmorphic disorder (BDD), and sleep difficulties are particularly prevalent during adolescence, the current study aims to examine associations between sleep quality and BDD symptoms among two separate samples of adolescents. It was hypothesized that lower reported sleep quality would be associated with higher reported BDD symptoms, even after controlling for anxiety and depression. Study 1 included 325 adolescents aged 11–18 years who completed an online survey via social media recruitment. The survey included questionnaires on BDD symptoms, sleep quality, and anxiety and depressive disorder symptoms. Study 2 included 396 adolescents who completed the same online survey. In study 1, a significant omnibus regression model accounted for 20.0% of the variance in adolescent BDD symptoms. After controlling for anxiety and depressive symptoms, adolescent sleep quality was significantly associated with BDD symptoms, with a small effect size (*sr*^*2*^ = 0.02). Study 2 yielded similar results, with a significant omnibus regression model accounting for 26.5% of the variance in adolescent BDD symptoms, and significant associations between adolescent sleep quality and BDD symptoms yielding a small effect size (*sr*^*2*^ = 0.01). Exploratory analyses in both studies demonstrated associations between difficulty returning to wakefulness and BDD symptoms. Adolescent sleep quality and BDD symptoms were linked, such that adolescents who reported lower quality sleep also reported higher levels of BDD symptoms. Future research can aim to replicate and extend these results using more rigorous (e.g., longitudinal, experimental) methods.

## Introduction

Body dysmorphic disorder (BDD) is a psychological disorder that involves preoccupation with perceived defects in appearance that appear minimal or non-existent to others [3]. BDD is relatively common, with a lifetime prevalence rate of 2.4% [31]. Retrospective work among adults with BDD indicated that the average age of onset for BDD was 16.7 years, while the modal age of onset was 12–13 years [10]. Indeed, work with a community sample of 3,149 adolescents found that 1.7% of adolescents in the sample reported probable BDD, while 3.4% of the sample reported subclinical BDD [46]. Additionally, BDD symptoms appear to be distributed dimensionally among adolescents, and adolescents who have elevated BDD symptoms report distress and impairment, even in the absence of crossing a diagnostic threshold [6]. In light of evidence that BDD tends to be chronic [38], onsets during adolescence [37], and earlier onset is associated with higher levels of comorbidity [10], there is a need to identify factors that may be associated with BDD symptoms during adolescence.

Interestingly, emerging research suggests that sleep disturbances may be important to consider in terms of BDD symptoms during adolescence. Sleep disturbances are common across individuals with psychopathology [5]. For example, sleep disturbances have been consistently associated with depression [36], anxiety [21], and posttraumatic stress disorder [4], suggesting that sleep disturbances may represent a transdiagnostic factor associated with multiple psychological disorders and symptoms. Recently, a nascent literature has also implicated sleep disturbances in the development and maintenance of obsessive–compulsive disorder (OCD) and related disorders [22]. The obsessive compulsive and related disorders (OCRD) category was introduced in The Diagnostic and Statistical Manual of Mental Disorders, Fifth Edition (DSM-5, [3]) and includes OCD along with other disorders characterized by obsessive thoughts and compulsive behaviors. These other disorders include excoriation disorder, hoarding disorder, trichotillomania, and BDD.

Although little work to date has examined the associations between sleep disturbances and BDD specifically, emerging work has linked other OCRDs to alterations in sleep [22]. For example, compared to a group of healthy control participants, participants with OCD reported increased subjective (i.e., sleep quality, sleep onset latency, sleep efficiency, and daytime dysfunction) and objective sleep disturbances (i.e., shorter sleep onset latency, lower total sleep time, lower sleep efficiency, [25]). Further, a meta-analysis indicated that OCD is related to disruptions in both the duration and timing of sleep [35]. Notably, research among clinical samples demonstrated that 92% of youth with pediatric OCD reported a sleep-related problem, with 27.3% of youth with pediatric OCD reporting five or more sleep-related problems [50]. Additionally, a clinical sample of non-depressed children with OCD demonstrated significant reduced total sleep time and longer wake periods after sleep onset than a matched group of healthy control children [1]. Indeed, a recent review demonstrated that youth with OCD report greater subjective sleep disturbances (i.e., poorer sleep quality, reduced sleep duration and efficiency, worse daytime dysfunction, and higher numbers of sleep disturbances) than age matched healthy controls [44]. Beyond more general sleep disturbances, OCD symptoms have also been repeatedly linked to self-reported insomnia symptoms among adults. For example, after controlling for depressive symptoms, self-reported insomnia symptoms were cross-sectionally [51] and longitudinally [19, 20] associated with elevated OCD symptoms. In contrast, among a sample of adolescents, while OCD was prospectively related to increased self-reported insomnia symptoms, self-reported insomnia symptoms were not prospectively linked to increased OCD symptoms after controlling for baseline OCD symptoms [2]. This indicates that the associations between OCD symptomology and insomnia symptoms may vary as a function of developmental phase.

A small literature has examined the associations between OCRDs other than OCD and sleep disturbances. For example, a study among adults with trichotillomania and excoriation disorder indicated that adults with trichotillomania and excoriation reported significantly more sleep difficulties (i.e., total sleep problems, sleep quality, sleep disturbances, use of sleep medication, and daytime dysfunction) than a control sample of healthy adults, even after controlling for anxiety and depression symptoms [43]. Additionally, among a sample of 24 adults with hoarding disorder, insomnia symptoms were positively associated with hoarding symptom severity, even after controlling for diagnoses of depression and PTSD [40]. Taken together, these studies provide preliminary evidence that subjective sleep disturbances may be linked to OCRDs.

In the most relevant work to date, muscle dysmorphia symptoms among adolescents and young adults were cross-sectionally and prospectively associated with shorter self-reported sleep duration and difficulty falling asleep. Specifically, symptoms related to appearance intolerance, drive for size, and functional impairment were most strongly associated with sleep disturbances [27]. Further, among a sample of bodybuilders/fitness athletes (BoFA, *n* = 44) during COVID-19, BoFA with probable muscle dysmorphia reported significantly greater insomnia symptoms than BOFA without probable muscle dysmorphia [28]. Next, among a clinical sample of 66 adolescents with BDD, 48.5% of adolescents in the sample reported clinically significant insomnia symptoms [47]. Additionally, Sevilla-Cermeno et al. [47] found that adolescents with clinically significant insomnia symptoms reported more severe BDD symptoms, worse functioning in daily activities, higher rates of co-morbid depression, and were less likely to be considered treatment responders/remitters compared to you with BDD without clinically significant insomnia. In contrast, among a clinical sample of 77 adults with BDD, approximately two thirds of the sample reported elevated insomnia symptoms, although sleep disruption was not associated with BDD symptoms severity [8]. Additionally, results of Bernstein et al. [8] study suggested that pre-treatment sleep disruption was not associated with treatment progress, while sleep improvements were not associated with treatment improvement. Given the limited body of work that has examined how subjective sleep disturbances may be associated with BDD symptoms (and the mixed results of the existing work), there is a need to examine the association between subjective sleep disturbances and BDD symptoms among adolescents.

There are a number of reasons to think that subjective sleep disturbances may be associated with BDD symptoms during adolescence. Sleep disturbances during adolescence may present differently than sleep disturbances among adults. For example, between 9 and 9.25 h of sleep a night are required for optimal cognitive and emotional functioning among adolescents [26], [48]. Unfortunately, only one third of American high school students currently receive the recommended amount of sleep each night [7]. The “perfect storm model” of sleep during adolescence proposes that the maturation of bioregulatory mechanisms coincide with psychosocial factors to result in short, ill-timed, sleep [12, 23]. These changes coincide with earlier school start times over the course of middle school and high school leading to greater levels of adolescent sleep deprivation and daytime sleepiness [13]. Indeed, many adolescents experience a mismatch between circadian phase and environmental sleep demands leading to low quality sleep [53]. In light of evidence that adults with OCRD report lower sleep quality than healthy controls [33], and that low-quality sleep is associated with increased mental health symptoms among adolescents [29, 41], the current study aims to examine the associations between adolescent sleep quality and BDD symptoms.

Given the evidence reviewed above, the aim of the current study was to examine the associations between self-reported adolescent sleep quality and adolescent BDD symptoms among two separate samples of adolescents. A number of hypotheses guided the current investigation. First, in Study 1, it was hypothesized that adolescents who reported lower quality sleep would also report higher body dysmorphia symptoms (Hypothesis 1). Given that a large body of work has demonstrated that anxiety symptoms [29] and depressive symptoms [43] are associated with sleep quality during adolescence, as well as the association between negative emotionality and body dysmorphia symptoms [49], the current study included anxiety and depressive symptoms as covariates in current analyses. Next, the goal of Study 2 was to replicate the findings from Study 1 using a separate, independent sample of adolescents using an empirically valid assessment of adolescent BDD symptoms. As with Study 1, it was hypothesized that in Study 2, adolescent sleep quality would be negatively associated with adolescent body dysmorphia symptoms after controlling for anxiety and depressive symptoms (Hypothesis 2). Finally, post-hoc exploratory analyses were conducted in both Study 1 and Study 2 to explore the associations between ASWS subscales (i.e., going to bed, falling asleep, maintaining sleep, reinitiating sleep, and returning to wakefulness) and BDD symptoms.

## Study 1 Methods

### Participants and Recruitment

Participants for Study 1 were 325 adolescents between the ages of 11 and 18 years (*M*_*age*_ = 14.82 years, *SD* = 1.11 years). In Study 1, adolescents reported the following gender identities: 92 adolescents identified as boys (28.3%), 162 adolescents identified as girls (49.8%), 61 adolescents identified as non-binary/third gender (18.8%), and 10 adolescents (3.1%) did not provide data on their gender identity. In the current study adolescents were able choose more than one racial/ethnic identity leading to the following racial/ethnic background: Caucasian/White (75.1%), Hispanic/Latino (14.5%), Asian American (11.4%), Black/African American (6.2%), Native American (3.7%), and “Other” (4.9%). Unfortunately, no norms have been published using the RCADS to assess levels of nonbinary/third gender adolescent affective symptoms; therefore, this study does not report clinical elevations for youth who identified as nonbinary/third gender or youth who declined to report their gender identity. However, using *T*-scores and published norms [15], 26 adolescent boys and girls reported borderline clinically significant depressive symptoms, and 109 adolescent boys and girls reported clinically significant depression scores. Twenty-six adolescent boys and girls reported borderline clinically significant anxiety scores, and 83 adolescent boys and girls reported clinically significant generalized anxiety symptoms. Taken together, this suggests that 53.1% of the adolescent boys and girls in the current sample reported borderline or clinically significant depression, and 42.9% of adolescent boys and girls in the sample reported borderline or clinically significant total anxiety scores. Due to experimenter error described below in the measures section, we could not compute clinical BDD scores.

### Procedures

Adolescents were recruited through paid advertisements displayed on social media (i.e., Facebook and Instagram). Ads for the study were displayed between March and August of 2021. Methods of the recruitment strategy used for the current study have been described in more detail elsewhere [9]. Briefly, adolescents viewed an advertisement that contained a picture of adolescents on their cell phones, the text on the ad invited adolescents to participate in a study which aimed to “learn more about adolescent emotions.” The advertisement stated that the study had been approved by the institutional review board (IRB) at the institution. Consistent with prior work done in samples of adolescents recruited online [30], the IRB at the institution waived the requirement for parental consent. Prior to taking the survey, adolescents were informed that they could skip as many questions as they chose, and at the end of the survey, adolescents were offered an opportunity to enter into a raffle for the prospect to win a $20.00 Amazon gift card. The current study included a single attention check question (“what color is the sky”). In order to be included in the current study, adolescents had to provide the right answer to the attention check question. The attention check question was answered by 434 adolescents. Of the adolescents who answered the attention check question, 387 adolescents (89%) provided the correct answer. The 325 adolescents who passed the attention-check and completed necessary measures were included in the current study.

### Measures

**Demographics.** Adolescents provided information about their age, gender, grade, and race/ethnicity.

**Body image questionnaire, child and adolescent version (BIQ-C).** BDD symptoms were assessed using the Body Image Questionnaire Child and Adolescent Version (BIQ-C; [52]). The BIQ-C assesses a number of symptoms related to BDD (i.e., distress, appearance checking, impairment, and avoidance). At the start of the measure, participants are asked to complete an initial question querying if the adolescent has any appearance concerns. If the adolescent denied appearance concerns, they were given a total score of 0. If adolescents reported an appearance concern, they were then allowed to report up to five body areas of concern. They then responded to 12 items while thinking about their areas of bodily concern (e.g., “how often do you check your feature,” “how much do you feel like your appearance is the most important thing about you?”). Due to experimenter error, participants rated responses on a 1 (*Never/Not at all*) to 4 (*Always/A lot*) scale (instead of the standard 0 to 8 scale employed for this measure in most studies). Internal consistency in the current study was good (α = 0.88).

**Revised child anxiety and depression scale (RCADS).** Adolescent anxiety and depressive symptoms were assessed using the Revised Child Anxiety and Depression Scale (RCADS; [14]. The RCADS is a self-report measure that consists of 47-items. It assesses anxiety and depressive symptoms in children and adolescents and includes subscales for social anxiety disorder, panic disorder, separation anxiety disorder, obsessive compulsive disorder, generalized anxiety disorder, and major depressive disorder. A total anxiety score can also be computed reflecting overall anxiety symptoms. The current study employed the RCADS total anxiety subscale and the depression subscale. Consistent with previous work (Timpano et al. 2014), modified total RCADS depression and total anxiety scores were calculated by removing two sleep items from the depression scale (i.e., “I have trouble sleeping” and “I am tired a lot”) and one item from the anxiety scale (“I feel scared if I have to sleep on my own”). This was done in order to reduce spurious correlations between the RCADS depression and total anxiety subscales with adolescent sleep quality. Adolescents rated the frequency of which they experience the statements listed (e.g., “I feel worthless,” “I worry bad things will happen to me”) on a 0 (*Never*) to 3 (*Always*) Likert-type scale. The RCADS has good psychometric properties [15]. In the current sample, internal consistency was good to excellent (α_depression scale_ = 0.89, α_anxiety_ = 0.96).

**Adolescent sleep wake scale (ASWS).** Adolescent sleep quality was measured using the Adolescent Sleep Wake Scale (ASWS; [32]. The ASWS is a 28-item measure that consists of a total score (reflecting overall sleep quality) and five subscales: going to bed (“I have trouble making myself go to bed at bedtime”), falling asleep (“I have trouble going to sleep”), maintaining sleep (“During the night, I toss and turn in my bed”), reinitiating sleep (“After waking up during the night, I have trouble going back to sleep”), and returning to wakefulness (“In the morning, I wake up feeling rested and alert”). Adolescents read each item and rated how often they had experienced each problem on a six-point rating scale from 1 (*always*) to 6 (*never*). Lower scores indicate lower quality sleep. The ASWS has demonstrated adequate internal consistency and validity in adolescents (Le Bourgeois et al. 2005. In the current sample, internal consistency was acceptable to excellent (α_total_ = 0.91, α_goingtobed_ = 0.79; α_fallingasleep_ = 0.81; α_maintainingsleep_ = 0.82; α_reinitiatingsleep_ = 0.79; α_returningtowakefulness_ = 0.84).

### Analytic Approach

As a first step, assumptions of normality were tested. Next, descriptive statistics and correlations were computed. Third, two hierarchical multiple regression analyses were conducted. The model had two steps: (1) the main effect of adolescent anxiety and depressive symptoms were entered at step 1; and (2) in the next step adolescent sleep quality was entered simultaneously with adolescent anxiety and depressive symptoms. Next, a separate regression was conducted for exploratory analyses. The model had two steps: (1) the main effect of adolescent anxiety and depressive symptoms were entered at step 1; and (2) in the next step adolescent ASWS subscales (i.e., going to bed, falling asleep, maintaining sleep, reinitiating sleep, and returning to wakefulness) were entered simultaneously with adolescent anxiety and depressive symptoms. For the purposes of this investigation, *sr*^2^ was used as an index of effect size (0.01 = small, 0.09 = medium, and 0.25 = large; [16]. Bootstrapping was employed in all regression analyses.

## Study 1 Results

### Descriptive Statistics and Correlations

Descriptive information and zero order correlations are presented in Table 1. Consistent with expectations and prior literature, BDD symptoms were significantly positively correlated with adolescent anxiety and depressive symptoms, and negatively correlated with adolescent sleep quality. Adolescent sleep quality was significantly negatively correlated with adolescent anxiety and depressive symptoms. Adolescent anxiety and depressive symptoms were significantly positively associated with one another.

**Table 1:** Descriptive data and zero-order relations between relevant continuous variables

	M (SD) Study 1	Mean (SD) Study 2	1	2	3	4
1. BDD	20.21 (14.89)	24.96 (19.47)	–	−0.39***	0.41***	0.38***
2. ASWS Total	95.54 (22.34)	101.57 (21.73)	−0.39***	–	−0.60***	−0.61***
3. RCADS_Anxiety	49.30 (22.54)	45.22 (19.91)	0.49***	−0.56***	–	0.77***
4. RCADS_Depression	11.89 (5.76)	10.21 (5.19)	0.45***	−0.56***	0.79***	–

Correlations for Study 1 located in the top half of the table, correlations for study 2 located in the bottom half of the table. BDD = Body Dysmorphia Symptoms, ASWS = Adolescent Sleep Quality, RCADS_Anxiety = adolescent anxiety symptoms, RCADS_Depression = adolescent depression symptoms

** p < 0.01, *** *p* < 0.001 (two-tail)

### Primary Hypothesis Test

A significant omnibus regression model [*F* (3,324) = 26.94, *p* < 0.001] emerged that accounted for 20.1% of the variance in adolescent BDD symptoms. As noted in Table 2, in step 1, adolescent anxiety symptoms (*sr*^*2*^ = 0.03) and depressive symptoms (*sr*^*2*^ = 0.01) were significantly associated with adolescent BDD symptoms, these effects were small in magnitude. In step 2, after controlling for adolescent anxiety and depressive symptoms, adolescent sleep quality was a significant predictor of adolescent BDD symptoms, this effect was small in magnitude (*sr*^*2*^ = 0.02).

**Table 2:** Study 1: Adolescent sleep problems and anxiety as well as depression symptoms as predictors of body dysmorphia symptoms

			ΔR^2^	*B [95% CI]*	*β*	*SE*	*sr*^*2*^	*p*
*Dependent Variable: BDD Symptoms*								
Step 1			0.177***					
	RCADS_Anxiety			0.187 [.087, .288]	0.287	0.051	0.03***	< 0.001
	RCADS_Depression			0.408[.008, .808]	0.158	0.203	0.01*	0.046
Step 2			0.024**					
	RCADS_Anxiety			0.144 [.040, .247]	0.220	0.052	0.02**	0.007
	RCADS_Depression			0.220 [−0.192, .631]	0.085	0.209	0.00	0.295
	ASWS total			−0136 [−0.220, −0.051]	−0.206	0.045	0.02**	0.002

* BDD* Body Dysmorphia Symptoms;* ASWS* adolescent Sleep Wake Scale;* RCADS_Anxiety * Adolescent Anxiety Symptoms;* RCADS_Depression* adolescent Depression Symptoms

*p < 0.005, ** p < 0.01, *** *p* < 0.001 (two-tail)

### Exploratory Analyses

A significant omnibus regression model [*F* (7,324) = 11.87, *p* < 0.001] emerged that accounted for 20.8% of the variance in adolescent BDD symptoms. As noted in Table 3, in step 1, adolescent anxiety symptoms (*sr*^*2*^ = 0.03) and depressive symptoms (*sr*^*2*^ = 0.01) were significantly associated with adolescent BDD symptoms, these effects were small in magnitude. In step 2, the ASWS subscales (i.e., going to bed, falling asleep, maintaining sleep, reinitiating sleep, and returning to sleep) were added to the regression model. In this final step of the model, adolescent depressive symptoms were no longer associated with adolescent BDD symptoms. Additionally, after controlling for adolescent anxiety and depressive symptoms, returning to wakefulness was the only ASWS subscale associated with adolescent BDD symptoms, this effect was small in magnitude (*sr*^*2*^ = 0.01), and approached statistical significance (*p* = 0.053).

**Table 3:** Study 1: adolescent sleep problems subscales and anxiety as well as depression symptoms as predictors of body dysmorphia symptoms

		ΔR^2^	B [95% CI]	β	SE	sr^2^	p
*Dependent variable: BDD symptoms*							
Step 1		0.177***					
	RCADS_Anxiety		0.187 [0.087, 0.288]	0.287	0.051	0.03***	< .001
	RCADS_Depression		0.408 [0.008, 0.808]	0.158	0.203	0.01*	0.046
Step 2		0.031*					
	RCADS_Anxiety		0.152 [0.047, 0.258]	0.233	0.054	0.02**	0.005
	RCADS_Depression		0.184 [−0.238, 0.607]	0.071	0.215	0.00	0.391
	Going to bed		−0.224[−0.586, 0.138]	−0.080	0.184	0.00	0.225
	Falling asleep		−0.020 [−0.368,.327]	−0.009	0.177	0.00	0.908
	Staying asleep		−0.083 [−0.364, 0.199]	−0.037	0.143	0.00	0.564
	Reinitiating sleep		−0.112 [−0.429, 0.206]	−0.046	0.161	0.00	0.489
	Returning to Wakefulness		−0.340 [−0.684, 0.005]	−0.120	0.175	0.01	0.054

*BDD* Body dysmorphia symptoms; *ASWS* adolescent sleep wake scale; *RCADS_Anxiety* adolescent anxiety symptoms; *RCADS_Depression* adolescent depression symptoms

*p < 0.05, ** p < 0.01, *** *p* < 0.001 (two-tail)

## Study 2 Methods

### Participants, Recruitment, and Procedure

In order to ensure that the results of Study 1 replicated, the aim of Study 2 was the examine the associations between sleep quality and BDD symptoms among a separate sample of adolescents. Recruitment procedures for Study 2 were similar to the methods for Study 1. In Study 2, 549,717 participants were shown the advertisement. Next, 2104 individuals clicked on the advertisement and were taken to the survey. Advertisements for the study ran between February and March of 2023. The current study included two attention-check questions (“what color is the sky” and “click the option that says C”) that adolescents had to answer correctly in order to be included in Study 2. Four hundred and ninety-three adolescents answered the attention-check questions, and of these, 483 (98%) adolescents answered the question “what color is the sky” correctly. In terms of the second question, 450 (91%) adolescents answered the question “pick option C” correctly. The 396 adolescents who passed both attention-check questions and completed measures for the current study were included in the current study (*M*_*age*_ = 15.93 years*, SD* = 1.49 years).

In the current sample, 167 adolescents identified as boys (42.4%), 185 adolescents identified as girls (47.0%), and 42 adolescents identified as non-binary/third gender (10.7%). In the current study adolescents were able choose more than one racial/ethnic identity leading to the following racial/ethnic background: Caucasian/White (64.1%), Asian American (16.7%), Black/African American (13.4%), Hispanic/Latino (13.4%), Native American (5.8%), and “Other” (1.3%). As in Study 1, to the best of our knowledge, no norms have been published using the RCADS to assess levels of nonbinary/third gender adolescent affective symptoms; therefore, this study does not report clinical elevations for youth who identified as nonbinary/third gender or youth who declined to report their gender identity. However, using *T*-scores and published norms [15], 43 adolescent boys and girls reported borderline clinically significant depressive symptoms, and 95 adolescent boys and girls reported clinically significant depression scores. Forty-nine adolescent boys and girls reported borderline clinically significant anxiety scores, and 77 adolescent boys and girls reported clinically significant anxiety symptoms. Taken together, this suggests that 43.5% of the adolescent boys and girls in the current sample reported borderline or clinically significant depression, and 39.7% of adolescent boys and girls in the sample reported borderline or clinically significant total anxiety scores. In terms of BDD scores, based on the norms published by Schneider et al. [45] 26.5% of adolescent girls, and 45.9% of adolescent boys in the current sample reported elevated BDD symptoms.

### Measures

**Demographics.** Adolescents provided information about their age, gender, grade, and race/ethnicity.

**Body image questionnaire, child and adolescent version-9 item version (BIQ-C-9).** The Body Image Questionnaire Child and Adolescent Version-9 Item Version (BIQ-C-9; [45, 52] was used to assess adolescent BDD symptoms. The BIQ-C-9 is similar to the BIQ-C described in Study 1 (see full details above), but uses 9 items rather than 12. In previous work, the BIQ-C-9 has demonstrated good internal consistency in male (α = 0.86) and female (α = 0.89) adolescents, and has been shown to have superior model fit to the 12-item BIQ-C [45]. In Study 2, consistent with empirical precedent, participants rated responses on a 0 (*not at all distressing*) to 8 (*extremely distressing*) scale. Internal consistency in the current study was excellent (α = 0.90).

**Revised child anxiety and depression scale (RCADS).** See Study 1 for a complete description. In the current sample, internal consistency was good to excellent (α_depression scale_ = 0.87, α_anxiety_ = 0.95).

**Adolescent sleep wake scale (ASWS).** See Study 1 for a complete description. In the current sample, internal consistency was acceptable to excellent (α_total_ = 0.91; α_goingtobed_ = 0.72; α_fallingasleep_ = 0.77; α_maintainingsleep_ = 0.78; α_reinitiatingsleep_ = 0.79; α_returningtowakefulness_ = 0.84).

### Analytic Approach

The analytic approach for Study 2 was identical to the approach used in Study 1.

## Study 2 Results

### Descriptive Statistics and Correlations

Descriptive information and zero order correlations are presented in Table 1. BDD symptoms were significantly positively correlated with adolescent anxiety and depressive symptoms, and negatively correlated with adolescent sleep quality. Adolescent sleep quality was significantly negatively correlated with adolescent anxiety and depressive symptoms. Adolescent anxiety and depressive symptoms were significantly positively associated with one another.

### Primary Hypothesis Test-Replication of Study 1 Findings

Consistent with Study 1, a significant omnibus regression model [*F* (3,395) = 47.04, *p* < 0.001] emerged that accounted for 26.5% of the variance in adolescent BDD symptoms. As noted in Table 4, in step 1, adolescent anxiety symptoms (*sr*^*2*^ = 0.05) and depressive symptoms (*sr*^*2*^ = 0.01) were significantly associated with adolescent BDD symptoms, these effects were small in magnitude. In step 2, after controlling for adolescent anxiety and depressive symptoms, adolescent sleep quality was a significant predictor of adolescent BDD symptoms, this effect was small in magnitude (*sr*^*2*^ = 0.01).

**Table 4:** Study 2: Adolescent sleep problems and anxiety as well as depression symptoms as predictors of body dysmorphia symptoms

		ΔR^2^	B [95% CI]	β	SE	sr^2^	p
*Dependent variable: BDD symptoms*							
Step 1		0.252***					
	RCADS_Anxiety		0.362 [0.226, 0.497]	0.370	0.069	0.05***	< 0.001
	RCADS_Depression		0.585 [0.066, 1.04]	0.156	0.264	0.01*	0.027
Step 2		0.013**					
	RCADS_Anxiety		0.316 [0.178, 0.445]	0.323	0.070	0.03**	< 0.001
	RCADS_Depression		0.425 [−0.250, 0.689]	0.113	0.269	0.00	0.115
	ASWS total		−0.127 [−0.220, −0.032]	−0.142	0.048	0.01**	0.009

*BDD* Body dysmorphia symptoms; *ASWS* adolescent sleep wake scale; *RCADS_Anxiety* adolescent anxiety symptoms; *RCADS_Depression* adolescent depression symptoms

*p < 0.05, ** p < 0.01, *** *p* < 0.001 (two-tail)

### Exploratory Analyses

Consistent with Study 1, a significant omnibus regression model [*F* (7395) = 21.88, *p* < 0.001] emerged that accounted for 28.3% of the variance in adolescent BDD symptoms. As noted in Table 5, in step 1, adolescent anxiety symptoms (*sr*^*2*^ = 0.05) and depressive symptoms (*sr*^*2*^ = 0.01) were significantly associated with adolescent BDD symptoms, these effects were small in magnitude. In step 2, the ASWS subscales (i.e., going to bed, falling asleep, maintaining sleep, reinitiating sleep, and returning to sleep) were added to the regression model. In this final step of the model, adolescent depressive symptoms were no longer associated with adolescent BDD symptoms. Additionally, after controlling for adolescent anxiety and depressive symptoms, returning to wakefulness was the only ASWS subscale associated with adolescent BDD symptoms, this effect was small in magnitude (*sr*^*2*^ = 0.02).

**Table 5:** Study 1: Adolescent sleep problems subscales and anxiety as well as depression symptoms as predictors of body dysmorphia symptoms

		ΔR^2^	B [95% CI]	β	SE	sr^2^	p
*Dependent variable: BDD symptoms*							
Step 1		0.252***					
	RCADS_Anxiety		0.362 [0.226, 0.497]	0.370	0.069	0.05***	< 0.0001
	RCADS_Depression		0.585 [0.066, 1.04]	0.156	0.264	0.01*	0.027
Step 2		0.031**					
	RCADS_Anxiety		0.337 [0.192, 0.481]	0.344	0.074	0.04***	< 0.001
	RCADS_Depression		0.281 [−0.256, 0.817]	0.075	0.273	0.00	0.304
	Going to bed		−0.089 [−0.557, 0.380]	−0.022	0.238	0.00	0.711
	Falling asleep		0.150 [−0.297, 0.597]	0.046	0.227	0.00	0.509
	Staying asleep		−0.228 [−0.662, 0.206]	−0.068	0.221	0.00	0.301
	Reinitiating sleep		0.048 [−0.359, 0.450	0.014	0.206	0.00	0.824
	Returning to wakefulness		−0.642 [−0.998, −0.285]	−0.188	0.181	0.02***	< 0.001

*BDD* Body dysmorphia symptoms; *ASWS* adolescent sleep wake scale; *RCADS_Anxiety* adolescent anxiety symptoms; *RCADS_Depression* adolescent depression symptoms

*p < 0.05, ** p < 0.01, *** *p* < 0.001 (two-tail)

## Discussion

The aim of the current study was to extend the literature linking sleep disturbances to OCRDs by examining the association between sleep quality and BDD symptoms among two separate samples of adolescents. Results of both studies suggested that adolescent sleep quality was negatively associated with adolescent BDD symptoms, such that adolescents who reported lower quality sleep also tended to report higher levels of BDD symptoms. Post-hoc exploratory analyses expanded on these results, demonstrating that the only ASWS subscale associated with BDD symptoms was the “returning to wakefulness” subscale [this subscale approached significance (*p* = 0.540), in Study 1, and reached conventional levels of significance (*p* < 0.001) in Study 2]. It is noteworthy that these results were significant after controlling for both anxiety and depressive symptoms. Although the current data did not come from clinical samples, they extend prior research (e.g., [22] and provide preliminary evidence that BDD symptoms among youth may be associated with lower quality sleep. The current results suggest a number of important future research directions.

First, in both studies, the association between sleep quality and BDD symptoms maintained significance after controlling for both anxiety and depressive symptoms. This is partially consistent with other work examining the association between OCRDs and sleep [43, 51]. Both depression [36] and anxiety [21] symptoms have been identified as significant correlates of sleep difficulties, and tend to be highly comorbid with BDD symptoms during adolescence [34]. Interestingly, in the current study, only adolescent anxiety and adolescent sleep quality were significantly linked to adolescent BDD symptoms. In contrast, adolescent depressive symptoms were not associated with adolescent BDD symptoms. This was surprising in light of previous research suggesting that BDD symptoms are associated with depressive symptoms [39]. Notably adolescent depressive and anxiety symptoms evidenced strong correlations in the current study (*r* = 0.77–0.79*)*. Although the VIF (1.54–2.74) and tolerance values (0.36–0.38) in the current study were within an acceptable range [11], it is possible that the null depression result was due to multicollinearity. Therefore, moving forward, it will be important to replicate and refine knowledge of the association between BDD symptoms, anxiety symptoms, depressive symptoms, and sleep quality among youth. For example, future work could meaningfully extend the literature by examining the associations between sleep quality, affective symptoms, and BDD symptoms prospectively or using different measures of adolescent anxiety and depressive symptoms. Although the current study provides valuable initial support for an association between sleep quality and BDD symptoms, it is not possible to determine the directionality of these associations. Indeed, it is possible that given the cross-sectional nature of the current findings, BDD symptoms may interfere with adolescent sleep quality (c.f., adolescent sleep quality affecting BDD symptoms), or these associations may be bidirectional. Longitudinal approaches would allow researchers to examine the direction of these effects, and if sleep quality and BDD symptoms may be bidirectionally associated with one another. Additionally, prospective data collections would allow for more complex analytical models (e.g., longitudinal mediation models, [17]) designed to parse apart the associations between sleep quality, BDD symptoms, and internalizing symptoms over time.

Post-hoc exploratory analyses suggested that the only subscale of the ASWS that was associated with BDD symptoms among adolescents was the returning to wakefulness scale. The returning to wakefulness scale reflects how rested adolescents feel upon waking in the morning. The subscale includes items such as “In the morning, I wake up and feel ready to get up for the day” and “I have trouble getting out of bed in the morning.” The association between the returning to wakefulness scale approached significance in Study 1 and reached significance in Study 2, although its effect size was small in both studies. These results provide preliminary evidence that adolescents who report feeling less rested upon waking are also more likely to report higher BDD symptoms. Previous research among adolescents demonstrated adolescents with a circadian preference for eveningness (i.e., adolescents who prefer later bed and rise times) also report significantly more difficulty returning to wakefulness in the morning than adolescents who prefer earlier wake and bed times [53]. The exploratory results in the current study suggest that it may, therefore, be important to consider adolescent chronotype in relation to BDD symptoms. To the best of our knowledge, no work to date has examined the association between chronotype and BDD symptoms. In the most relevant work to date, although chronotype was not prospectively related to the development of OC symptoms among a sample of adolescents [2], chronotype was prospectively linked to OC symptoms after controlling for baseline OC and depressive symptoms among adults [9]. Therefore, and important next step in this line of work may be to examine if chronotype may be associated with BDD symptoms among both adolescents and adults. This may help elucidate results of the current study.

Next, moving forward, there is a need to better understand why sleep quality may be associated with BDD symptoms. Storch et al. [50] outlined a number of possible reasons that youth with OCRD symptoms may be more likely to report lower quality sleep than youth without OCRD symptoms including: heightened levels of anxiety/physiological arousal, engagement in rituals that may interfere with sleep onset, and more frequent cognitive/perseverative symptoms that may interfere with sleep. Reynolds et al. [42] expanded on this to propose a model of factors that may underlie the association between OCD and sleep difficulties in children. The model suggests that OC relevant nighttime routines/rituals may increase pre-sleep arousal, delay bedtime, and elicit parental accommodation thereby increasing sleep onset latency and restricting sleep [42]. This restricted sleep may lead to increased obsessions and emotional inhibition, which likely bidirectionally maintain one another. The degree to which such a model may apply to adolescent BDD remains an empirical question. Additionally, it is unclear how self-reported sleep quality may map onto these models. Therefore, moving forward, future research can usefully examine if similar processes may be at play among youth with BDD symptoms, and if youth with BDD symptoms evidence similar alterations in sleep to youth with other OCRDs using more objective measures (i.e., actigraphy, polysomnography) and other self-report measures (e.g., insomnia measures).

There were a number of limitations to the current study. The first limitation is the generalizability of the findings in the present samples. It is possible that both study samples were subject to self-selection bias, as participants were recruited via social media, and there were notably fewer participants engaging in the studies than the number of individuals who received ads for the studies and who clicked on the survey links. Additionally, given the rising popularity of using social media to recruit adolescents [24], it will be important to improve knowledge of the strengths (e.g., increased access to adolescents) and potential weaknesses (e.g., limited oversight of survey completion) of this recruitment approach. Second, the samples for both studies consisted of primarily White adolescents. Future research should examine the generalizability of these findings to non-White samples. Third, the current work relied primarily on self-report assessments of sleep. Moving forward, it will be important to utilize objective sleep measures (e.g., actigraphy, polysomnography) to assess associations between BDD symptoms and sleep among adolescents. Relatedly, it is important to note that while the results of the current study are statistically significant, the effect sizes are small. It is possible that the significant results in the current study may be attributed to shared method variance [17]. Replicating the current findings using objective sleep measures will help clarify the nature of the association between sleep and BDD symptoms during adolescence. Additionally, future research should consider exploring more specific areas of sleep disturbances beyond general sleep quality (e.g., insomnia symptoms, sleep onset latency, sleep efficiency, [22], as these may highlight more specific associations between sleep disturbances and body dysmorphia symptoms. Fourth, the current sample consisted of primarily non-clinical populations of adolescents. Although previous work indicates that BDD symptoms are distributed dimensionally among adolescents [6], moving forward, it will be important to examine these associations in clinical samples, using structured clinical interviews. Indeed, between 10 and 50% of adolescents in the current sample reported borderline or clinically elevated affective symptoms, moving forward, the current associations should be replicated among a larger sample of clinical adolescents. In particular, in Study 2, the number of boys in the current sample that reported elevated BDD symptoms was notably high. Clinical cutoff scores suggested by Schneider et al. [45] were used to calculate BDD elevations, but it is important to note that the threshold for meeting elevation is lower for adolescent boys (a score of 25) than girls (a score of 41). Moving forward, it will be imperative to examine the associations between sleep quality and BDD symptoms using more rigorous assessment methods to ensure current findings replicate. Next, the current study did not examine the role of OCD symptoms in the association between sleep quality and BDD symptoms. This is a notable limitation as BDD and OCD symptoms often co-occur [18]. Moving forward, studies can usefully expand on the current findings by carefully considering the role OCD symptoms may play in the association between sleep quality and BDD symptoms. Finally, in Study 1, due to experimenter error, participants responded to the BIQ-C on a 0 to 4 scale (c.f., a 0–8 scale). In order to address this concern, Study 2 used the appropriate scaling for the BIQ-C-9. Nonetheless, this is a notable limitation of the current work, and future work should endeavor to replicate current findings using appropriate scaling.

## Data Availability

Data will be made available upon request.
